# High Diversity of Genogroup I Picobirnaviruses in Mammals

**DOI:** 10.3389/fmicb.2016.01886

**Published:** 2016-11-23

**Authors:** Patrick C. Y. Woo, Jade L. L. Teng, Ru Bai, Annette Y. P. Wong, Paolo Martelli, Suk-Wai Hui, Alan K. L. Tsang, Candy C. Y. Lau, Syed S. Ahmed, Cyril C. Y. Yip, Garnet K. Y. Choi, Kenneth S. M. Li, Carol S. F. Lam, Susanna K. P. Lau, Kwok-Yung Yuen

**Affiliations:** ^1^Department of Microbiology, The University of Hong KongHong Kong, China; ^2^State Key Laboratory of Emerging Infectious Diseases, The University of Hong KongHong Kong, China; ^3^Research Centre of Infection and Immunology, The University of Hong KongHong Kong, China; ^4^Carol Yu Centre for Infection, The University of Hong KongHong Kong, China; ^5^Collaborative Innovation Center for Diagnosis and Treatment of Infectious Diseases, The University of Hong KongHong Kong, China; ^6^Ocean Park CorporationHong Kong, China

**Keywords:** diversity, picobirnaviruses, mammals, genogroup I, sea lion

## Abstract

In a molecular epidemiology study using 791 fecal samples collected from different terrestrial and marine mammals in Hong Kong, genogroup I picobirnaviruses (PBVs) were positive by RT-PCR targeting the partial RdRp gene in specimens from five cattle, six monkeys, 17 horses, nine pigs, one rabbit, one dog, and 12 California sea lions, with 11, 9, 23, 17, 1, 1, and 15 sequence types in the positive specimens from the corresponding animals, respectively. Phylogenetic analysis showed that the PBV sequences from each kind of animal were widely distributed in the whole tree with high diversity, sharing 47.4–89.0% nucleotide identities with other genogroup I PBV strains based on the partial RdRp gene. Nine complete segment 1 (viral loads 1.7 × 10^4^ to 5.9 × 10^6^/ml) and 15 segment 2 (viral loads 4.1 × 10^3^ to 1.3 × 10^6^/ml) of otarine PBVs from fecal samples serially collected from California sea lions were sequenced. In the two phylogenetic trees constructed using ORF2 and ORF3 of segment 1, the nine segment 1 sequences were clustered into four distinct clades (C1–C4). In the tree constructed using RdRp gene of segment 2, the 15 segment 2 sequences were clustered into nine distinct clades (R1–R9). In four sea lions, PBVs were detected in two different years, with the same segment 1 clade (C3) present in two consecutive years from one sea lion and different clades present in different years from three sea lions. A high diversity of PBVs was observed in a variety of terrestrial and marine mammals. Multiple sequence types with significant differences, representing multiple strains of PBV, were present in the majority of PBV-positive samples from different kinds of animals.

## Introduction

Picobirnaviruses (PBVs) are small non-enveloped bisegmented double-stranded RNA viruses found in human and a wide variety of mammals and birds. Since its first discovery in fecal samples of humans and rats in Pereira et al. (1988a,b), PBVs have been reported in a variety of other terrestrial mammals, birds and environmental water samples (Fregolente et al., 2009; Ghosh et al., 2009; Symonds et al., 2009; Martinez et al., 2010; Ganesh et al., 2011a; Malik et al., 2011; Wang et al., 2012; Bodewes et al., 2013; Gillman et al., 2013; Ng et al., 2014; Ribeiro et al., 2014; Zhang et al., 2014). In 2012, we reported the discovery of a PBV, named otarine PBV (Ot-PBV), in a California sea lion (*Zalophus californianus*) in Hong Kong, which was the first PBV reported in a marine mammal (Woo et al., 2012). Recently, we have also described the first discovery and a diversity of PBVs in dromedary camels from the Middle East (Woo et al., 2014b).

The genome of PBV consists of two segments named segment 1 and segment 2. Segment 1 contains the capsid gene and another open reading frame which encodes for a putative protein of unknown function, whereas segment 2 contains the RNA-dependent RNA polymerase (RdRp) gene (Wakuda et al., 2005; Woo et al., 2012). By sequence and phylogenetic analyses, PBVs are classified into genogroups I and II based on the RdRp gene sequence (Rosen et al., 2000; Wakuda et al., 2005). Recently, it has also been reported that novel genogroups of PBVs have been detected in human and environmental samples (Smits et al., 2014; Zhang et al., 2015). As of December 31 2015, 931 PBV nucleotide sequences have been submitted to GenBank. However, only nine are complete/near-complete segment 1 sequences and 21 are complete/near-complete segment 2 sequences. Among these nine complete/near-complete segment 1 and 21 complete/near-complete segment 2 sequences, 18 are genogroup I sequences.

Our recent study on dromedary camel PBVs (Woo et al., 2014b) and our preliminary analysis using the limited PBV nucleotide sequences in GenBank (data not shown) revealed that different genogroup I PBVs could be present in different animals of the same species. Since a high diversity of genogroup I PBVs may exist in different animals, we performed a molecular epidemiology study using fecal samples collected from different terrestrial and marine mammals in Hong Kong. In addition, we studied the evolution of genogroup I PBVs in California sea lions by serially collecting their fecal samples for 6 years and sequenced and analyzed the complete segments 1 and 2 of the PBV-positive samples.

## Materials and Methods

### Terrestrial and Marine Mammal Surveillance and Sample Collection

This study was performed in strict accordance with local ordinance and the recommendations by the Committee on the Use of Live Animals in Teaching and Research (CULATR) at The University of Hong Kong. All specimens of bats, monkeys, cats, and dogs were collected with the assistance of the Department of Agriculture, Fisheries and Conservation, Hong Kong Special Administrative Region (HKSAR); those of pigs and cattle were collected with the assistance of the Department of Food, Environmental and Hygiene, HKSAR, from various locations in HKSAR; and those of horses were collected with the assistance of the Hong Kong Jockey Club. All specimens of rabbits were collected from live food animal markets in Guangzhou, China, in October 2007. Rectal swabs were collected using procedures described previously (Lau et al., 2005). All fecal samples of marine mammals, including Indo-Pacific bottlenose dolphins, California sea lions and harbor seals, were collected by veterinary surgeons of the Ocean Park in HKSAR (Woo et al., 2014a). A total of 791 samples collected over a 75-month period (October 2007 to December 2013) from 157 bats, 52 monkeys, 100 pigs, 58 cats, 58 dogs, 50 cattle, 106 rabbits, 95 horses, 46 Indo-Pacific bottlenose dolphins, 54 California sea lions, and 15 harbor seals were tested (**Table 1**).

**Table 1:** Prevalence of PBVs in mammals by RT-PCR targeting a partial fragment of RdRp gene.

Animal type	Scientific name	Number of samples obtained	Number (%) of samples positive for genogroup I PBVs	Health status
**Terrestrial mammals**				
Cattle	NA	50	6 (12.0)	Deceased
Monkey^∗^	NA	52	6 (11.5)	Healthy
Horse^†^	NA	95	17 (18.0)	Sick with fever
Pig	NA	100	9 (9.0)	Deceased
Rabbit	*Oryctolagus cuniculus*	106	1 (0.9)	Healthy
Dog	*Canis familiaris*	58	1 (2.0)	Healthy
Cat	*Feline domestica*	58	0 (0)	Healthy
Bat	*Hipposideros pomona*	103	0 (0)	Healthy
	*Rhinolophus affinis*	14	0 (0)	Healthy
	*Rhinolophus sinicus*	40	0 (0)	Healthy
**Marine mammals**				
California sea lion	*Zalophus californianus*	54	12 (22.0)	Healthy
Indo-Pacific bottlenose dolphin	*Tursiops aduncas*	46	0 (0)	Healthy
Harbor seal	*Phoca vitulina*	15	0 (0)	Healthy


*^∗^Most monkeys found in our locality are hybrids of *Macaca mulatta* and *Macaca fascicularis.*^†^All horses included in this study were thoroughbred racehorses stabled at the Hong Kong Jockey Club.*

*NA, not available.*

### RNA Extraction

Viral RNA was extracted from rectal and cloacal swabs and fecal samples using EZ1 Virus Mini Kit v2.0 (Qiagen, Germany). RNA was eluted in 60 μl of AVE buffer (Qiagen, Germany) and about 200 ng of RNA was used as template for RT-PCR.

### RT-PCR for PBVs and DNA Sequencing

Genogroup I PBV screening was performed by PCR amplification of a 205-bp fragment of the RdRp gene of genogroup I PBVs using conserved primers (5′-CAAARTTYGACCARCACTT-3′ and 5′-TCRTCDGCRTTGGTACCACC-3′) designed by multiple alignments of the available RdRp genes of PBVs. Reverse transcription was performed using the SuperScript III kit (Invitrogen, USA) and the reaction mixture (10 μl) contained RNA, first-strand buffer (50 mM Tris-HCl pH 8.3, 75 mM KCl, 3 mM MgCl_2_), 5 mM DTT, 50 ng random hexamers, 500 μM of each dNTPs and 100 U Superscript III reverse transcriptase. The mixtures were incubated at 25°C for 5 min, followed by 50°C for 60 min and 70°C for 15 min. The PCR mixture (25 μl) contained cDNA, PCR buffer (10 mM Tris-HCl pH 8.3, 50 mM KCl, 2 mM MgCl_2_), 200 μM of each dNTPs and 1.0 U *Taq* polymerase (Applied Biosystems, USA). The mixtures were amplified in 60 cycles of 94°C for 1 min, 50°C for 1 min and 72°C for 1 min and a final extension at 72°C for 10 min in an automated thermal cycler (Applied Biosystems, USA).

All PCR products were gel-purified using the QIAquick gel extraction kit (Qiagen, Germany). Both strands of the PCR products were sequenced twice with an ABI Prism 3730xl DNA Analyzer (Applied Biosystems, USA), using the two PCR primers. As multiple nucleotide peaks were observed in most sequencing results, it was suggested that more than one type of PBV were present in each sample, thus the purified PCR products were cloned into the pCR-II-TOPO TA cloning vector (Invitrogen, USA) according to manufacturer’s instructions. Both strands of 10 clones for each sample were sequenced, using primers 5′-TAATACGACTCACTATAGGG-3′ and 5′-CGGCTCGTATGTTGTGTGGA-3′. The sequences of the clones were compared with known sequences of the RdRp of PBVs in the GenBank database.

### Complete Segments 1 and 2 Sequencing of Genogroup I Otarine PBVs

Nine complete segments 1 and 15 complete segment 2 of otarine PBVs were amplified and sequenced using published strategies for double-stranded RNA viruses (Attoui et al., 2000), using RNA extracted from the original specimens of sea lions positive for PBV as template. Viral RNA was extracted using the EZ1 virus mini kit (Qiagen, Germany). Adaptor primer, with 3′ NH_2_ blocking group, was ligated to the 3′ termini of the viral RNA and subjected to reverse transcription using complementary primer. After RNA hydrolysis, reannealing and end-filling, single-primer amplification of viral genomic segments was performed using complementary primer and genome specific primers. The 5′ and 3′ ends of the viral genomes were confirmed by rapid amplification of cDNA ends using the 5′/3′ RACE kit (Roche, Germany). The PCR products were gel purified and sequenced using an ABI Prism 3700 DNA analyzer (Applied Biosystems, USA). Sequences were assembled and manually edited to produce the final sequences of the viral genomes.

### Genome Analysis

The nucleotide sequences of the genomes and the deduced amino acid sequences of the ORFs were compared to those of other PBVs. Novel genes were further predicted by FGENESV (SoftBerry, Inc.^1^), a trained pattern/Markov chain-based viral gene prediction program. Phylogenetic tree construction was performed using the maximum likelihood method and MEGA7 (Kumar et al., 2016), with bootstrap values being calculated from 1,000 trees. The optimal substitution model for each ORF was selected by MEGA7. Protein domain, family and functional site analyses were performed using ScanProsite (De Castro et al., 2006). Transmembrane and coiled-coil domains were predicted by TMHMM Server v 2.0 (Krogh et al., 2001) and COILS (Lupas et al., 1991) respectively.

### Quantitative RT-PCR

For real-time quantitative PCR assays, cDNA were amplified in SYBR Green I fluorescence reactions (Roche, Germany). Briefly, 10 μl of reaction mixtures containing 1 μl cDNA, 10 μl FastStart DNA master SYBR green I mix reagent (Roche) and 5 mM each of forward and reverse specific primers were thermal-cycled at 95°C for 10 min followed by 45 cycles of 95°C for 10 s, 60°C for 10 s and 72°C for 20 s using a Roche LightCycler 96 real time PCR system (Roche, Germany). Specific primers for each PBV segment were designed based on the sequences of all the segment 1 and segment 2 detected in the positive samples (**Table 3**). Plasmids with the corresponding target sequences were used for generating the standard curve. At the end of the assay, PCR products were subjected to a melting curve analysis (65–95°C, 0.1°C/s) to confirm the specificity of the assay.

## Results

### Detection of Diverse Genogroup I PBVs in Animals

**FIGURE 1:**
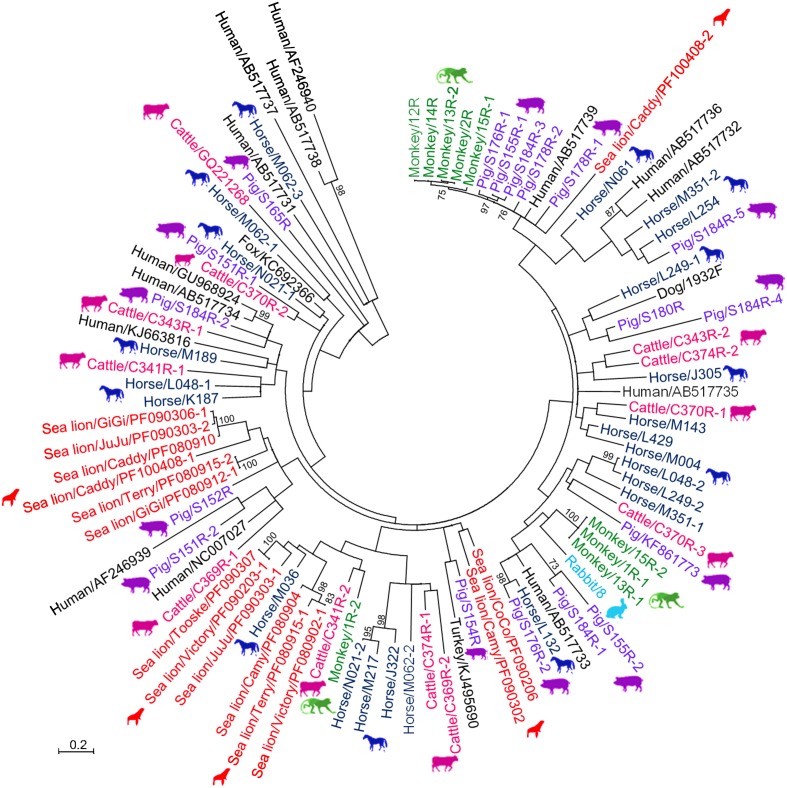
**Phylogenetic analysis of partial RdRp gene of genogroup I PBVs discovered in the present study.** The tree was constructed using the maximum-likelihood method and the optimal substitution model of T92+G+I, and rooted with genogroup II human strains, shown in italics. One hundred and ninety one nucleotide positions were included in the analysis. Bootstrap values below 70% are not shown. The scale bar indicated the number of nucleotide substitutions per site. All PBV strains discovered in this study are colored, with those detected in the same host highlighted in the same color. If more than one sequence type was found in the same sample, each sequence type was numbered in the order of identification (e.g., Monkey/13R-1, Monkey/13R-2, and Monkey/13R-3 indicated that there were three sequence types found in the same monkey sample 13R). All the accession numbers are given as cited in GenBank.

A total of 791 fecal specimens from 676 terrestrial mammals and 115 marine mammals were obtained (**Table 1**). RT-PCR for a 205-bp fragment in the RdRp gene of genogroup I PBVs was positive in specimens from six cattle, six monkeys, 17 horses, nine pigs, one rabbit, one dog, and 12 sea lions. Marked nucleotide polymorphisms were observed in most of the RdRp sequences, suggesting the possible existence of multiple strains in the same specimen. Therefore, the PCR products were cloned and 10 clones from each specimen were sequenced. Multiple sequence types were confirmed to be present in most samples. Sequence analysis of these clones revealed that there were 11, 9, 23, 17, 1, 1, and 15 sequence types in the positive specimens from the five cattle, six monkeys, 17 horses, nine pigs, one rabbit, one dog, and 12 sea lions respectively, and 47.4–89.0% nucleotide identities were observed between these clones and the corresponding sequences of other genogroup I PBV strains available in the GenBank database (**Figure 1**). The PBV sequences from each kind of animal were widely distributed in the whole phylogenetic tree with high diversity (**Figure 1**). No PBV was detected in the specimens obtained from the 58 cats, 157 bats, 46 Indo-Pacific bottlenose dolphins, and 15 harbor seals (**Table 1**).

### Otarine PBVs Complete Segments 1 and 2 Sequence Analysis

**FIGURE 2:**
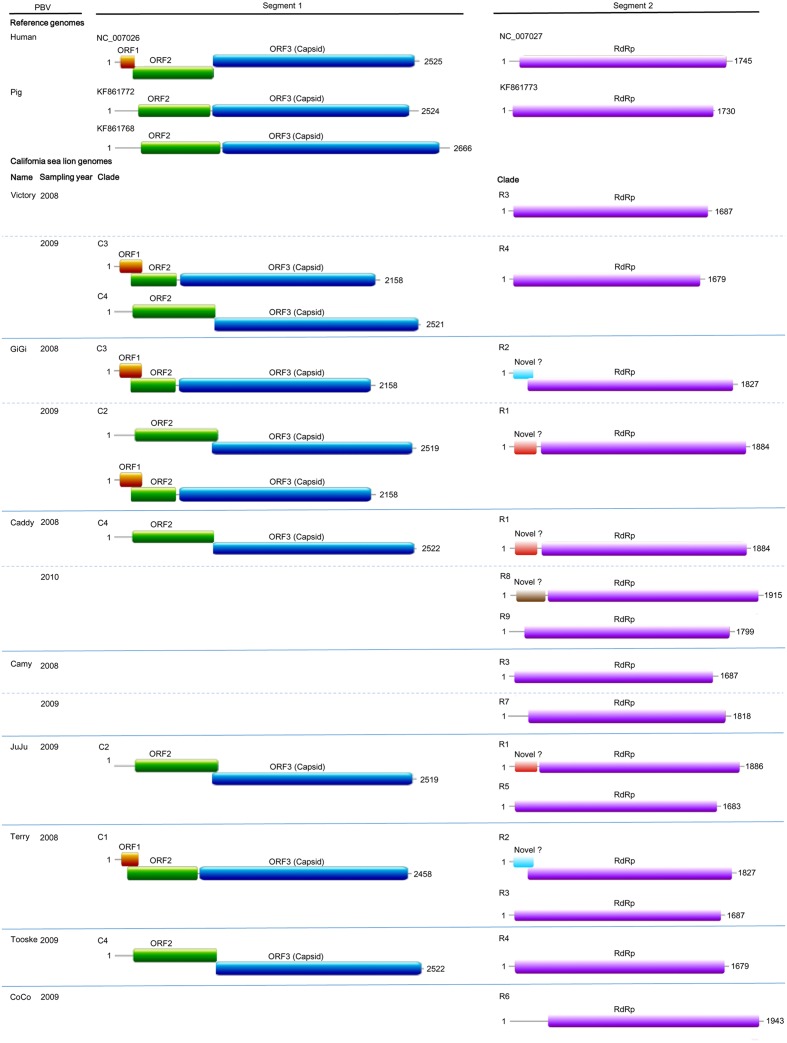
**Genome organization of otarine PBVs and the two representative genogroup I PBVs.** The name of each California sea lion, year of sample collection and clade (C1–C4 and R1–R9) for each otarine PBV are indicated. For the nine segment 1 sequences, the capsid protein is represented by a blue box. Upstream to the capsid protein, ORF1 and ORF2 are represented by orange and green boxes respectively. For the 15 segment 2 sequences, the RdRp is represented by a purple box. The three different kinds of previously undescribed ORF upstream to the RdRp in six of the 15 segment 2 are represented by boxes highlighted with different colors.

Nine complete segment 1 of otarine PBVs from sea lions were sequenced and assembled (**Table 2**; **Figure 2**). These segment 1 sequences ranged from 2,158 to 2,522 bases in length with overall G+C contents of 41.1–46.0%. The 5′ non-coding regions (44–169 bases) were AU-rich (G+C contents of 22.7–37.9%) with five conserved bases, GUAAA, located at the 5′ end. A predicted highly stable stem loop structure found in other known PBVs was observed in five segment 1 sequences as a result of the pairing of 5′-GUAAA-3′ and 5′-UUUAC-3′ in the 5′ non-coding region (Nates et al., 2011). The 3′ non-coding regions (19–32 bases) contained G+C contents ranging from 53.1 to 71.4% and end with 3–4 conserved bases (CTC, CTTC, or CACC). All the nine segment 1 sequences possess one long ORF (1,590–1,728 bp) encoding the capsid protein of 529–575 amino acids. These capsid proteins shared low (19.3–37.2%) amino acid identities with those of other PBV strains, being most closely related to turkey PBV TK/MN/2011 (GenBank number KJ495689), fox PBV F5-1 (GenBank number KC692367) and human PBV Hy005102 (GenBank number NC_007026). Upstream to the ORF for the capsid protein, there were one to two short ORFs in the nine segment 1, consistent with the organization of the segment 1 in other known PBVs (Wakuda et al., 2005; Bodewes et al., 2013; Banyai et al., 2014; Verma et al., 2015). The protein encoded by the ORF2 of segment 1 from the nine otarine PBVs possessed different numbers of repetitions of the same motif, ExxRxNxxxE, that was also observed in the corresponding protein in other known PBVs (Da Costa et al., 2011).

**Table 2:** Genomic features and coding potential of otarine PBVs detected in California sea lions in this study.

California sea lion	Sampling year	Segment 1						Segment 2					
			
		Clade	ORFs	Location (nt)	Length (nt)	Length (aa)	Frame	Clade	ORFs	Location (nt)	Length (nt)	Length (aa)	Frame
Victory	2008							R3	RdRp	45–1643	1599	532	3
	2009	C3	ORF1	45–230	186	61	3	R4	RdRp	46–1635	1590	529	1
			ORF2	136–510	375	124	1						
			ORF3	537–2126	1590	529	3						
	2009	C4	ORF2	164–835	672	223	2						
			ORF3	832–2502	1671	556	1						
GiGi	2008	C3	ORF1	45–230	186	61	3	R2	Novel ORF	39–185	147	48	3
			ORF2	136–510	375	124	1		RdRp	167–1783	1617	538	2
			ORF3	537–2126	1590	529	3						
	2009	C2	ORF2	170–847	678	225	2	R1	Novel ORF	46–210	165	54	1
			ORF3	850–2487	1638	545	1		RdRp	251–1846	1596	531	2
	2009	C3	ORF1	45–230	186	61	3						
			ORF2	136–510	375	124	1						
			ORF3	537–2126	1590	529	3						
Caddy	2008	C4	ORF2	165–836	672	223	3	R1	Novel ORF	46–210	165	54	1
			ORF3	833–2503	1671	556	2		RdRp	251–1846	1596	531	2
	2010							R8	Novel ORF	47–262	216	71	2
									RdRp	285–1874	1590	529	3
	2010							R9	RdRp	140–1759	1620	539	2
Camy	2008							R3	RdRp	46–1644	1599	532	1
	2009							R7	RdRp	168–1772	1605	534	3
JuJu	2009	C2	ORF2	170–847	678	225	2	R1	Novel ORF	48–212	165	54	3
			ORF3	850–2487	1638	545	1		RdRp	253–1848	1596	531	1
	2009							R5	RdRp	46–1638	1593	530	1
Terry	2008	C1	ORF1	55–198	144	47	1	R2	Novel ORF	39–185	147	48	3
			ORF2	104–688	585	194	2		RdRp	167–1783	1617	538	2
			ORF3	703–2430	1728	575							
	2008						1	R3	RdRp	46–1644	1599	532	1
Tooske	2009	C4	ORF2	165–836	672	223	3	R4	RdRp	46–1635	1590	529	1
			ORF3	833–2503	1671	556	2						
CoCo	2009							R6	RdRp	296–1897	1602	533	2


Fifteen complete segment 2 from the otarine PBVs were sequenced and assembled (**Table 2**; **Figure 2**). These segment 2 sequences ranged from 1,679 to 1,943 bases in length with overall G+C contents of 39.5–48.1%. The 5′ non-coding regions (38–295 bases) were also AU-rich (G+C contents of 18.4–35.9%) with the same conserved bases, GUAAA, located at the 5′ end. The stable stem loop structure observed in segment 1 was also observed in the 5′ non-coding regions of 13 segment 2 sequences. The 3′ non-coding regions (38–46 bases) have G+C contents ranging from 31.6 to 56.1% and end with four conserved bases (CUGC) in most of the genomes. All the 15 segment 2 sequences possess one long ORF (1,590–1,620 bp) encoding the RdRp of 529–539 amino acids. These RdRp shared 44.5–70.6% amino acid identities with those of other genogroup I PBV strains, being most closely related to fox PBV F5-1 (GenBank number KC692366), human PBV 1-CHN-97 (GenBank number AF246939), human PBV GPBV6C1 (GenBank number AB517731), human PBV HuPBV-E-CDC16 (GenBank number KJ663816) and human PBV VS10 (GenBank number GU968924). They possess three conserved motifs (D-T/S-D, SG-T, GDD) commonly found in the RdRp sequences of other dsRNA viruses. Conserved cysteine and proline residues present in other genogroup I PBVs were also observed in all 15 segment 2 sequences. In contrast to the segment 2 sequences of other known PBVs, six of our 15 sequenced segment 2 possess a previously undescribed ORF 48–71 amino acids upstream to the RdRp ORF. Multiple alignments of the sequences of these six ORFs showed that they formed three groups that were not homologous to each other, with three ORFs belonging to the first group, two ORFs to the second group and one ORF to the third group (**Figure 2**). In the first group, the three ORFs showed 97.6–99.4% nucleotide identities among each other. In the second group, the two ORFs showed 100% nucleotide identity. Sequence analysis of these ORFs did not reveal any significant sequence homology to other proteins in the GenBank database. Protein sequence analysis also did not reveal any significant matches to other known protein domains, families or functional sites in the PROSITE database. Moreover, there are no transmembrane and coiled-coil domains predicted by sequence analyses in these protein sequences.

### Phylogenetic Analysis of Otarine PBVs Complete Segments 1 and 2 Sequences

**FIGURE 3:**
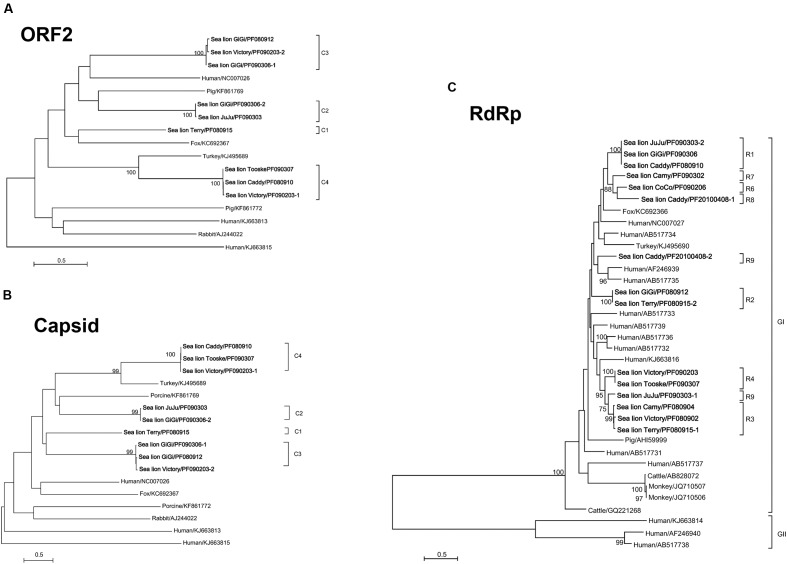
**Phylogenetic analyses of ORF2** **(A)**, capsid **(B)**, and RdRp proteins of otarine PBVs **(C)**. The trees are constructed using maximum likelihood method and the optimal substitution models of LG+G+F for ORF2 and LG+G for capsid and RdRp proteins. Two hundred and seventeen, 545 and 475 amino acid positions in ORF2, capsid and RdRp proteins were included respectively in the analyses. The scale bar indicated the number of nucleotide substitutions per site. Bootstrap values below 70% are not shown. The otarine PBVs characterized in this study are bolded. If more than one sequence type was found in the same sample, each sequence type was numbered in the order of identification (e.g., Victory/PF090203-1 and Victory/PF090203-2 indicated that there were two sequence types found in the same California sea lion sample Victory/PF090203). The clade of each otarine PBV is also indicated. All the accession numbers are given as cited in GenBank. GI and GII represent genogroup I and genogroup II PBVs respectively.

In the phylogenetic trees constructed using ORF2 (**Figure 3A**) and ORF3 (capsid protein) (**Figure 3B**), the nine sequenced segment 1 of the otarine PBVs were clustered into four distinct clades (C1–C4) (**Figures 3A,B**). The sequence types that belonged to each clade were identical for both trees, suggesting that there was no recombination between different PBVs. In the phylogenetic tree constructed using the RdRp gene, the 15 sequenced segment 2 of the otarine PBVs were clustered into nine distinct clades (R1–R9) (**Figure 3C**). Although, R1, R6, R7, and R8 as well as R3, R4, and R5 seemed to be further clustered in the tree, there were >14% amino acid difference between any two clades.

### Quantitative RT-PCR

Quantitative RT-PCR showed that the amounts of otarine PBV RNA ranged from 1.7 × 10^4^ to 5.9 × 10^6^ (for segment 1) and 4.1 × 10^3^ to 1.3 × 10^6^ (for segment 2) copies/ml in the fecal samples (**Table 3**).

**Table 3:** Quantitative RT-PCR assays of the California sea lions positive for PBV.

Name of California sea lion	Sample number	Segment 1/2 – sequence type	RNA concentration (copies/ml)	Sampling dates (YYYYMMDD)	Forward primer (5′–3′)	Reverse primer (5′–3′)
Victory						
	PF080902	Segment 2	2.4 × 10^4^	20080911	CCATGACTCAAGTCCTTACTAGT	TTTAGTGATCCGCTGGTCGACGGCA
	PF090203	Segments 1-1	1.4 × 10^6^	20090209	GAAGATGGTTATACCATTAGTGGTTA	AACTGTGAAGGAATTTTGAATCTG
	PF090203	Segments 1-2	2.2 × 10^4^	20090209	GAAGATTGTATGACCATGGGCGGTGA	AATGAACGTAACATTCATAATCTG
	PF090203	Segment 2	1.9 × 10^4^	20090209	TCGCTTCCGATATGACTCAA	ACATCTTAGTGATACGCTGG
Camy						
	PF080904	Segment 2	9.8 × 10^4^	20080911	CCATGACTCAAGTCCTTACTAGT	TTTAGTGATCCGCTGGTCGACGGCA
	PF090302	Segment 2	5.4 × 10^4^	20090304	ACTAGCCACGGTCTGGAGAT	ACACACACCATCTTGCCGAT
Caddy						
	PF080910	Segment 1	1.7 × 10^4^	20080912	GAAGATGGTTATACCATTAGTGGTTA	AACTGTGAAGGAATTTTGAATCTG
	PF080910	Segment 2	4.1 × 10^3^	20080912	GGTTTGGACGCATATCAAGA	ACATTCTAGTTATAGCTCGA
	PF100408	Segment 2-1	7.3 × 10^4^	20100425	AGATTGCTGTACCAGGAGCG	GCGCTACCATCTTAGGACCC
	PF100408	Segment 2-2	6.9 × 10^5^	20100425	TGGGTAGTGGTTCAGGAGGT	CGGTTATACCGGGGTACGTG
CoCo						
	PF090206	Segment 2	7.4 × 10^5^	20090217	GGTTGTGTTACCAGGAGCGA	CTTAATTGCCGCAGAGCGAC
GiGi						
	PF080912	Segment 1	9.7 × 10^4^	20080912	GAAGATTGTATGACCATGGGCGGTGA	AATGAACGTAACATTCATAATCTG
	PF080912	Segment 2	3.3 × 10^5^	20080912	GGAGATGACGTGGCACAGG	ATAGACGAGTTATCGCTTTA
	PF090306	Segment 1-1	5.9 × 10^6^	20090310	GAAGATTGTATGACCATGGGCGGTGA	AATGAACGTAACATTCATAATCTG
	PF090306	Segment 1-2	2.2 × 10^6^	20090310	GAAGATTTTGCAATTATGAGCGGTGA	AATAGTACGAGTGTTTGCAATCTG
	PF090306	Segment 2	1.3 × 10^6^	20090310	GGTTTGGACGCATATCAAGA	ACATTCTAGTTATAGCTCGA
Terry						
	PF080915	Segment 1	4.6 × 10^6^	20080912	GAAGATATGGCTATTATGAGTGGTGA	CCAGATTGTAGCATTTTCGATCTG
	PF080915	Segment 2-1	7.8 × 10^5^	20080912	CCATGACTCAAGTCCTTACTAGT	TTTAGTGATCCGCTGGTCGACGGCA
	PF080915	Segment 2-2	1.2 × 10^5^	20080912	GGAGATGACGTGGCACAGG	ATAGACGAGTTATCGCTTTA
JuJu						
	PF090303	Segment 1	1.0 × 10^6^	20090304	GAAGATTTTGCAATTATGAGCGGTGA	AATAGTACGAGTGTTTGCAATCTG
	PF090303	Segment 2-1	4.2 × 10^5^	20090304	GCATCTACTATGACCCAGGTT	ACATCTTAGTGATCCGCCGA
	PF090303	Segment 2-2	6.2 × 10^4^	20090304	GGTTTGGACGCATATCAAGA	ACATTCTAGTTATAGCTCGA
Tooske						
	PF090307	Segment 1	2.1 × 10^5^	20090318	GAAGATGGTTATACCATTAGTGGTTA	AACTGTGAAGGAATTTTGAATCTG
	PF090307	Segment 2	2.1 × 10^4^	20090318	TCGCTTCCGATATGACTCAA	ACATCTTAGTGATACGCTGG


### Longitudinal Detection of Otarine PBVs and Genome Evolution

Fecal samples were serially obtained from 18 sea lions over 6 years (**Table 4**). In the 12 samples that were positive for PBV, segment 2 could be detected and sequenced in all 12 samples, but segment 1 could only be detected and sequenced in seven samples due to difficulties in designing PCR primers as a result of the limited number of segment 1 PBV sequences available in GenBank. Overall, samples positive for PBV were collected mainly in 2008 and 2009. Among these positive results, most clades of segment 1 and segment 2 were observed in samples from either 2008 or 2009, while only segment 1 clades C3 and C4 and segment 2 clade R1 were present in samples from both 2008 and 2009. More than one clade of segment 2 was present in three of the 12 samples and more than one clade of segment 1 was present in two of the seven segment 1 positive samples. In four sea lions (Victory, Camy, Caddy, and GiGi), PBVs were detected in samples collected from two different years (**Table 4**). Among these four sea lions, the same segment 1 clade (C3) was present in two consecutive years in one sea lion (GiGi), with a total of 18 nucleotide changes in the capsid coding region of the same clade (C3) detected between 2008 and 2009. In the other three sea lions (Victory, Camy and Caddy), different clades were present in the fecal samples obtained in different years.

**Table 4:** Prevalence of PBVs identified in serial fecal samples of California sea lions.

Name of California sea lion	Detection of PBV (clade^∗^ for each otarine PBV is indicated in bracket)			
	
	2008	2009	2010	2013
Beamer	-	-	-	NA
Victory	+ (R3)	+ (C3, C4, R4)	-	-
BoyBoy	-	-	-	NA
Camy	+ (R3)	+ (R7)	-	NA
Wahoo	-	-	-	NA
Baja	-	-	NA	NA
Bobo	-	-	-	NA
Caddy	+ (C4, R1)	-	+ (R8, R9)	NA
Coco	-	+ (R6)	-	-
GiGi	+ (C3, R2)	+ (C2, C3, R1)	NA	NA
Julius	-	-	NA	NA
Jumanji	-	-	NA	-
Terry	+ (C1, R2, R3)	-	NA	NA
Beeper	-	-	-	-
JuJu	-	+ (C2, R1, R5)	NA	-
Jellybean	NA	-	NA	-
Tooske	NA	+ (C4, R4)	-	-
Julie	NA	-	NA	-


*^∗^C1–C4 and R1–R9.*

*+, positive for PBVs; -, negative for PBVs.*

*NA, samples not available.*

### Nucleotide Sequence Accession Numbers

The genome sequences of otarine PBVs obtained from the present study were deposited in GenBank with accession numbers KU729746–KU729769.

## Discussion

A high diversity of PBVs was observed in a variety of terrestrial and marine mammals. Despite the relatively high evolutionary rate of RNA viruses, those that infect a specific host usually fall into several discrete viral species; for example, human coronaviruses that infect human include four distinct species, namely OC43 (Vabret et al., 2003), 229E (Yeager et al., 1992), NL63 (Hofmann et al., 2005), and HKU1 (Woo et al., 2005). Viruses infecting a specific host do not form a “continuous” spectrum. However, when we tried to perform phylogenetic analysis on short fragments of PBVs amplified from human samples downloaded from the GenBank, it was noted that these human PBVs formed a spectrum covering the whole phylogenetic tree (data not shown). In the present study on PBVs of different animal hosts, a similar phenomenon was observed. PBVs from horses, pigs, and cattle were widely distributed in the whole phylogenetic tree, and PBVs from sea lions and monkeys also showed high diversity (**Figure 1**). In addition no two PBVs were detected to be the same among the 52 positive samples, except for that found in two pairs of sea lions, which possessed identical sequences even in the short fragment of 205 bases used for screening. The reason for this phenomenon remains unclear, although it may be partly due to the high mutation rate of PBVs, as quasispecies are frequently found in PBV sequences. Unfortunately, no PBV has been isolated in all studies so far. Therefore, further confirmation by repeated passage of a PBV strain and sequencing, which will determine its mutation rate more accurately, is not possible.

Multiple strains of PBV were present in the majority of PBV-positive samples from different kinds of animals. In the literature, the presence of multiple strains of PBV has only been described in fecal samples collected from human (van Leeuwen et al., 2010; Ganesh et al., 2011b), pigs (Bányai et al., 2008; Chen et al., 2014), chickens (Ribeiro et al., 2014), monkeys (Wang et al., 2012) and buffalos (Malik et al., 2014), despite the identification of PBVs in 24 different animals. In this study, multiple strains of PBV were observed in the same fecal samples of cattle, monkeys, horses, pigs, and sea lions. This phenomenon was also confirmed by sequencing the complete segments 1 and 2 of otarine PBVs directly from the fecal samples of sea lions (**Figure 2**; **Table 4**). Among the 12 samples that showed positive results, more than one clade of segment 2 were present in three of the 12 samples and more than one clade of segment 1 were present in two of the seven segment 1 positive samples (**Table 4**). It is of note that due to the low number of complete segments 1 and 2 sequences of PBV in GenBank, some of the segments in the fecal samples of the sea lions in the present study could not be amplified and sequenced. In fact, only nine complete/near-complete PBV segment 1 sequences are available in GenBank, making sequencing of segment 1 particularly difficult using the genome walking approach. Despite these technical difficulties, more than one segment 1 and/or more than one segment 2 were observed in at least five fecal samples of the sea lions in this study. The presence of more than one segment 1 and more than one segment 2 in the same sample is rare in other segmented RNA viruses. This phenomenon makes it difficult to ascertain which segment 1 corresponds to which segment 2 in individual PBV genomes from a specific fecal sample.

In the PBV genomes sequenced in this study, only the two ORFs that encode for the capsid protein and RdRp showed significant homologies to the corresponding ORFs encoding the same proteins in other PBVs. In addition to these two ORFs, an ORF1 and an ORF2 were found upstream to the ORF that encodes for the capsid protein in segment 1 of some PBVs. Bioinformatics analysis showed that the deduced amino acid sequences of ORF1 and ORF2 possess no significant homology with any known protein and no putative transmembrane domain was found. Interestingly, three different kinds of previously undescribed ORFs not homologous to each other were also found upstream to the ORF encoding RdRp in the segment 2 sequences of six otarine PBVs in this study (**Figure 2**). Similar to ORF1 and ORF2, these three ORFs were predicted by the gene prediction program FGENESV, instead of only the ORF Finder. These three kinds of ORFs showed no homology to any known proteins and did not possess any known protein domains, families or functional sites. Further experiments will be required to determine the functions of these three ORFs as well as ORF1 and ORF2.

**FIGURE 4:**
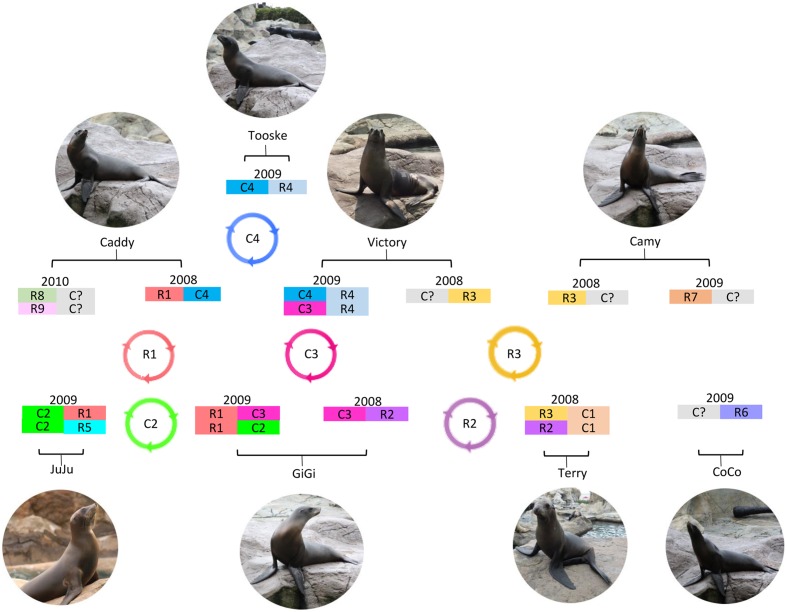
**Circulation and evolution model of otarine PBVs in California sea lions.** Possible otarine PBVs detected in the serial fecal samples of California sea lions collected in different years are shown. Possible reassortment of segments 1 and 2 of different clades (C1–C4 and R1–R9) circulating in the California sea lions are also indicated.

Picobirnaviruse probably evolves through mechanisms similar to other segmented RNA viruses (**Figure 4**). The most thoroughly studied segmented RNA viruses are the influenza viruses (negative-sense single-stranded RNA virus) and rotaviruses (double-stranded RNA virus). Influenza viruses evolve through reassortment of RNA segments resulting in antigenic shifts and RNA mutations leading to antigenic drifts and causing major pandemics, lots of mortalities and morbidities, and economic lost (Smith et al., 2009). As for rotaviruses, virus strains belonging to the same group can also reassort their genomes resulting in enormous diversity (Kirkwood, 2010). This provides one of the mechanisms for the emergence of new rotavirus strains leading to disease outbreaks and loss of vaccine efficacy (Kirkwood, 2010). The presence of multiple segment 1 and segment 2 in the same animal observed in this study provides the PBV genome a good opportunity for reassortment (**Figure 4**). In addition, in the serially collected fecal samples from sea lions, it was also observed that two segment 1 sequences that belonged to the same clade (C3) can be present in the same animal (GiGi) from two consecutive years (**Table 4**). There were 18 nucleotide changes in these two segment 1 sequences, leading to 11 amino acid changes. This could be due to persistence of the same PBV with mutational changes over 2 years or re-infection by another otarine PBV with segment 1 of the same clade but of a different sequence type.

In this study, a high diversity of PBVs was observed in a variety of terrestrial and marine mammals. Multiple sequence types with significant differences, representing multiple strains of PBV, were present in the majority of PBV-positive samples from different kinds of animals. These results suggest that PBV probably evolves through mechanisms similar to other segmented RNA viruses.

## Author Contributions

PW conceived of the study, designed the study, contributed reagents and drafted the manuscript. JT conceived of the study, designed the study, participated in data analysis and drafted the manuscript; RB carried out the molecular lab work and participated in data analysis. AW and AT participated in data analysis; PM and S-WH contributed reagents; CCL, SA, CY, GC, KL, and CSL carried out the lab work. SL revised the manuscript and contributed reagents; K-YY conceived of the study, designed the study, contributed reagents and revised the manuscript. All authors gave final approval for publication.

## Conflict of Interest Statement

The authors declare that the research was conducted in the absence of any commercial or financial relationships that could be construed as a potential conflict of interest.

## Footnotes

1 www.softberry.com/
